# Racial Disparity and Social Determinants in Receiving Timely Surgery Among Stage I–IIIA Non-small Cell Lung Cancer Patients in a U.S. Southern State

**DOI:** 10.3389/fpubh.2021.662876

**Published:** 2021-06-02

**Authors:** Paige Neroda, Mei-Chin Hsieh, Xiao-Cheng Wu, Kathleen B. Cartmell, Rachel Mayo, Jiande Wu, Chindo Hicks, Lu Zhang

**Affiliations:** ^1^Department of Public Health Sciences, Clemson University, Clemson, SC, United States; ^2^Louisiana Tumor Registry, School of Public Health, Louisiana State University Health Sciences Center, New Orleans, LA, United States; ^3^Department of Genetics, School of Medicine, Louisiana State University Health Sciences Center, New Orleans, LA, United States

**Keywords:** non-small cell lung cancer, timely surgery, racial disparity, social determinants, cancer registry

## Abstract

Delayed surgery is associated with worse lung cancer outcomes. Social determinants can influence health disparities. This study aimed to examine the potential racial disparity and the effects from social determinants on receipt of timely surgery among lung cancer patients in Louisiana, a southern state in the U.S. White and black stage I–IIIA non-small cell lung cancer patients diagnosed in Louisiana between 2004 and 2016, receiving surgical lobectomy or a more extensive surgery, were selected. Diagnosis-to-surgery interval >6 weeks were considered as delayed surgery. Social determinants included marital status, insurance, census tract level poverty, and census tract level urbanicity. Multivariable logistic regression and generalized multiple mediation analysis were conducted. A total of 3,616 white (78.9%) and black (21.1%) patients were identified. The median time interval from diagnosis to surgery was 27 days in whites and 42 days in blacks (*P* < 0.0001). About 28.7% of white and 48.4% of black patients received delayed surgery (*P* < 0.0001). Black patients had almost two-fold odds of receiving delayed surgery than white patients (adjusted odds ratio: 1.91; 95% confidence interval: 1.59–2.30). Social determinants explained about 26% of the racial disparity in receiving delayed surgery. Having social support, private insurance, and living in census tracts with lower poverty level were associated with improved access to timely surgery. The census tract level poverty level a stronger effect on delayed surgery in black patients than in white patients. Tailored interventions to improve the timely treatment in NSCLC patients, especially black patients, are needed in the future.

## Introduction

Lung cancer is the leading cause of cancer death in the United States (U.S.), accounting for 24% of all cancer deaths (1). In 2020, it is estimated that up to 228,820 new cases and 135,720 deaths from lung cancer occurred in the U.S (2). Non-small cell lung cancer (NSCLC) represents about 85% of all lung cancer cases (3). Survival of NSCLC patients has improved with the advancement of early detection and treatment (4). Surgical resection is the primary recommended treatment for patients with stage I-IIIA NSCLC (5). Timely care is an important indicator of the quality of care recommended by the Institute of Medicine (5). Delayed surgery is associated with tumor upstaging and worse survival (4, 6). Patients who receive surgery tend to have a longer wait time than those not receiving surgery, because of the multiple staging studies and preoperative examinations that are required (7, 8). It is of public health importance to examine the timeliness of NSCLC surgery.

Racial disparities exist in NSCLC diagnosis, treatment, and outcomes. Black patients are 16% less likely to be diagnosed at an early stage, 57% less likely to receive guideline concordant treatment, and 19% less likely to receive surgical treatment, when compared to their white counterparts. The survival rate is also lower in black patients than in white patients (9–11). From 2010 to 2015, Louisiana ranked 7th among states in the U.S. for lung cancer incidence and mortality rate (12). Blacks make up approximately 1/3 of the population in Louisiana, and have worse health status than other racial groups (10). Black residents in Louisiana have a higher lung cancer incidence (68.9 vs. 61.1 per 100,000) and poorer survival compared to black people in the U.S. on average (13, 14). Additionally, 17.9% of black NSCLC patients in Louisiana received early diagnosis while 21.4% of whites received early diagnosis (10). Thus, Louisiana NSCLC patients are the appropriate population to investigate the racial disparity in timely surgery of NSCLC.

Social determinants of health are societal factors that contribute to one's overall health or the health of the community (15). It has been argued throughout history that racial health disparities are not caused by biological difference; rather they are influenced by societal factors (16). Previous studies have found that social and economic factors influence disparities in lung cancer incidence and survival, even more than biological differences. Several social determinants, including income, insurance status, marital status, and rural residence, are associated with the receipt of standard care for NSCLC (17). This study had three objectives: (1) to investigate whether there is a racial disparity in receipt of timely surgery among stage I–IIIA NSCLC patients; (2) if the racial disparity exists, to examine whether the disparity can be explained by multiple social determinants; and 3) to explore whether the social determinants have a differential effect on timely surgery in each racial group.

## Methods

### Data Source and Study Population

The Louisiana Tumor Registry (LTR) is a population-based state cancer registry and a participant of the National Cancer Institute's Surveillance, Epidemiology, and End Results (SEER) program and the National Program of Cancer Registries of the Centers for Disease Control and Prevention. LTR routinely collects data on the characteristics, diagnosis, and the first course treatment of newly diagnosed cancers among Louisiana residents. This study identified white and black patients who were diagnosed with stage I-IIIA NSCLC between 2004 and 2016 from the LTR database. Only patients who received surgery of lobectomy or more extensive surgery were included. Patients who received neoadjuvant treatment, or who had unknown timing of treatment initiation were excluded.

### Variables

The main exposure variable was race (white, black). The outcome variable was timely receipt of surgery. As we included stage I–IIIA patients to whom the recommended first definitive treatment was surgery, and excluded the patients who received neoadjuvant treatment and those whose timing of treatment initiation was unclear, we considered that surgery was the first treatment for the selected patients. The main guidelines for the timing of treatment initiation of NSCLC patients is to receive surgery within 6 weeks, as specified by the RAND corporation (18), within 8 weeks by the British Thoracic Society (19), and within 4–8 weeks by the American College of Chest Physicians (20). Thus, we used ≤ 6 weeks as a middle representation across these guidelines (≤ 6 weeks, >6 weeks).

Social determinants examined in this study included type of insurance (private, Medicare, Medicaid, no insurance, unknown), marital status (married, single or divorced or widowed or other, unknown), census tract level population under the federal poverty level (<10%, 10–19.9%, ≥20%), and census tract level urbanicity (urban, rural). Census tract level urbanicity was determined based on the Census Bureau's identification of urban and rural areas. Other covariates included age at diagnosis (<54, 55–64, 65–74, ≥75), sex, tumor histology (adenocarcinoma, other), tumor size (<3 cm, 3–7 cm, >7 cm), American Joint Committee on Cancer (AJCC) stage (I, II, IIIA), grade (well-differentiated, moderately differentiated, poorly differentiated/undifferentiated, unknown), lymph node involvement status (negative, positive), surgery type (resection of lob, resection extended), and comorbidity (Charlson Comorbidity Index score of 0, 1, 2+) (21).

### Statistical Analysis

We used Chi-square test to compare the categorical variables. Students' *t*-test and Wilcoxon-Mann-Whitney test were applied to compare the mean and median time interval between tumor diagnosis and surgical resection by race. We applied three logistic regression models to examine the racial difference in receiving timely surgery. In Model 1, we employed a crude model to examine the racial disparity for delayed surgery. In Model 2, we controlled for clinical factors, including age, sex, comorbidity, histology type, tumor size, AJCC stage, tumor grade, lymph node involvement status, and surgery type, to examine whether the adjusted odds ratio (OR) of delayed surgery remained significant for race. As a final step, social determinants were adjusted in Model 3 to examine whether the racial disparity could be explained. To examine the effect of social determinants on delayed surgery in each racial group, a stratified analysis was conducted among white and black patients. These analyses were performed using SAS 9.4 (SAS Institute Inc.), and statistical tests of significance were based on a 2-sided test with significance levels of 0.05.

**Figure 1:**
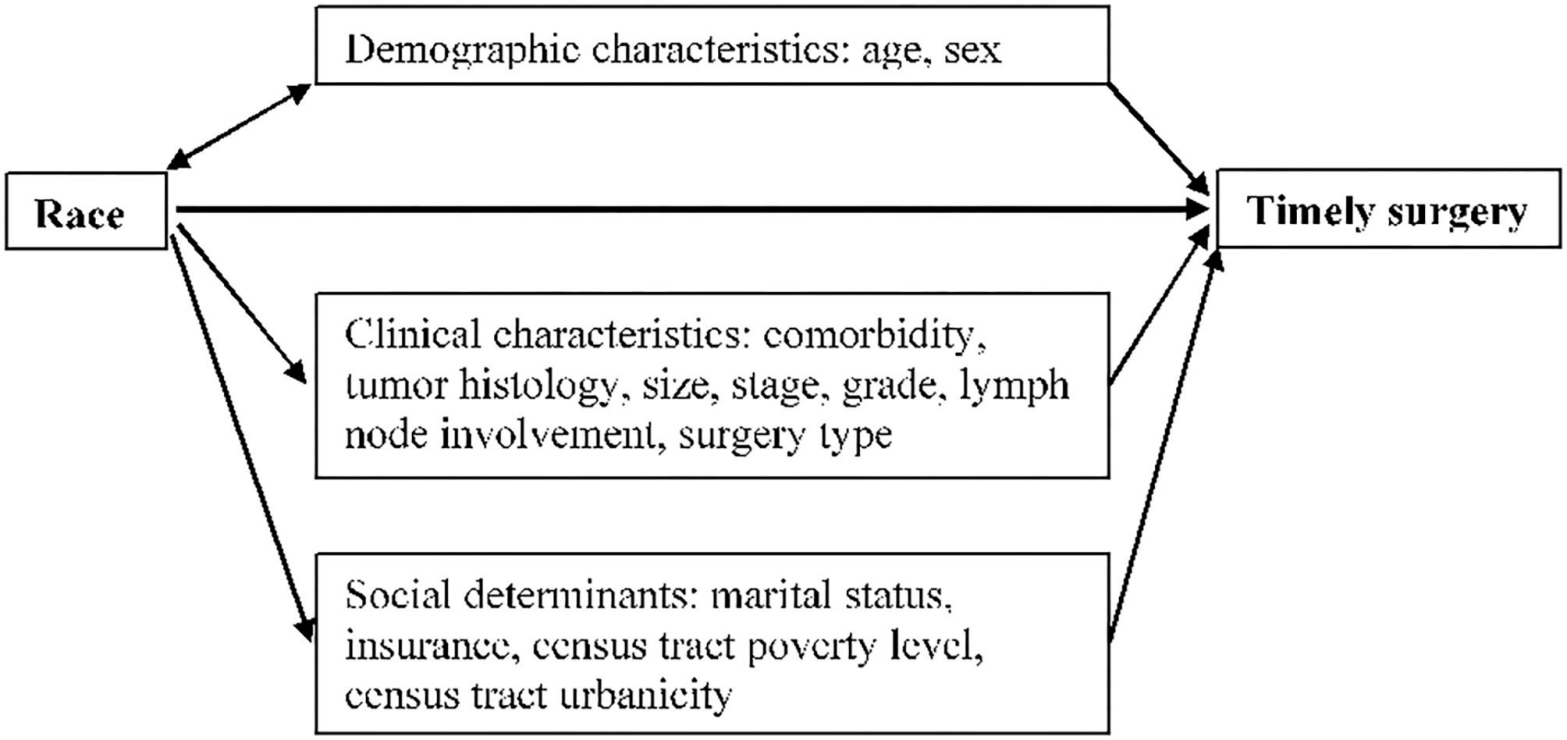
Conceptual diagram of racial disparity in receiving timely surgery for non-small cell lung cancer patients.

To examine the percentage of racial differences in receiving timely surgery which was explained by the social determinants, we conducted general multiple mediation analysis (MMA) to evaluate the mediating effects. MMA evaluates the mediating effects under the counterfactual framework, which can report joint mediation effects through multiple selected mediators simultaneously, considering the correlation among these mediators (22). As shown in the conceptual diagram in Figure 1, race was treated as exposure variable and timely surgery as outcome variable. Demographic characteristics (age, sex) were deemed as potential confounders. Clinical factors (comorbidity, tumor histology, size, stage, grade, lymph node involvement, and surgery type) and social determinants (marital status, insurance, census tract poverty level, and census tract urbanicity) were considered as groups of mediators in the pathway between race and timely surgery. The total effect of race on timely surgery was the sum of the direct effect from race and the indirect effect through each group of mediators (total effect = direct effect + indirect effect) (22). The percentage of the total effect explained by each group of mediators was calculated as the ratio of the indirect effect divided by the total effect. We used R version 4.0.0 with mma package to conduct the mediation analysis. The 95% confidence interval was obtained by bootstrap with 500 repetitions.

## Results

A total of 3,616 patients, including 2,854 (78.9%) white and 762 (21.1%) black patients were included in our study (Table 1). White patients were more likely to be older than 65 years, married, have stage I or lymph node negative disease, while black patients were more likely to be covered by Medicaid, live in a census tract with higher urbanicity and poverty level. For both racial groups, over half of patients were male, without comorbid conditions, diagnosed with adenocarcinoma and <3 cm tumors, or receiving lobectomy. The median time interval from diagnosis to surgery was 27 days for whites and 42 days for blacks (*P* < 0.0001). About 28.7% of white patients and 48.4% of black patients received surgery after 6 weeks from diagnosis (*P* < 0.0001).

**Table 1:** Characteristics of Louisiana stage I–IIIA non-small cell lung cancer patients by race, 2004–2016, *n* (%).

	**Total**	**White**	**Black**	***P*-value**
*N*	3,616	2,854 (78.9)	762 (21.1)	
**Demographic factors**				
Age				<0.0001
<54	496 (13.7)	353 (12.4)	143 (18.8)	
55–64	980 (27.1)	722 (25.3)	258 (33.9)	
65–74	1463 (40.5)	1176 (41.2)	287 (37.7)	
75+	677 (18.7)	603 (21.1)	74 (9.7)	
Sex				0.47
Female	1,913 (52.9)	1,501 (52.6)	412 (54.1)	
Male	1,703 (47.1)	1,353 (47.4)	350 (45.9)	
**Clinical factors**				
Tumor histology				0.09
Adenocarcinoma	2,186 (60.5)	1,705 (59.7)	481 (63.1)	
Non-adenocarcinoma	1,430 (39.5)	1,149 (40.3)	281 (36.9)	
Tumor size				0.84
<3 cm	2,149 (59.4)	1,702 (59.6)	447 (58.7)	
3–7 cm	1,289 (35.6)	1,014 (35.5)	275 (36.1)	
>7 cm	178 (4.9)	138 (4.9)	40 (5.3)	
AJCC stage				0.02
Stage I	2,447 (67.7)	1,964 (68.8)	483 (63.4)	
Stage II	750 (20.7)	568 (19.9)	182 (23.9)	
Stage IIIA	419 (11.6)	322 (11.3)	97 (12.7)	
Lymph node involvement				0.02
Negative	2,758 (76.3)	2,202 (77.2)	556 (73.0)	
Positive	858 (23.7)	652 (22.9)	206 (27.0)	
Grade				0.64
Well-differentiated	420 (11.6)	335 (11.7)	85 (11.2)	
Moderately differentiated	1,467 (40.6)	1,170 (41.0)	297 (39.0)	
Poorly/undifferentiated	1,449 (40.1)	1,129 (39.6)	320 (42.0)	
Unknown	280 (7.7)	220 (7.7)	60 (7.9)	
Surgery type				0.67
Resection of lobe	3,200 (88.5)	2,529 (88.6)	671 (88.1)	
Resection extended	416 (11.5)	325 (11.4)	91 (11.9)	
Comorbidity score				0.05
0	2,000 (55.3)	1,587 (55.6)	413 (54.2)	
1	1,053 (29.1)	844 (29.6)	209 (27.4)	
2+	563 (15.6)	423 (14.8)	140 (18.4)	
**Social determinants**				
Marital status				<0.0001
Married	2,111 (58.4)	1,798 (63.0)	313 (41.1)	
Single/divorced/widowed/other	1,395 (38.6)	968 (33.9)	427 (56.0)	
Unknown	110 (3.0)	88 (3.1)	22 (2.9)	
Insurance				<0.0001
Private	1,347 (37.3)	1,100 (38.5)	247 (32.4)	
Medicare/other public	1,682 (46.5)	1,390 (48.7)	292 (38.3)	
Medicaid	409 (11.3)	245 (8.6)	164 (21.5)	
No insurance	104 (2.9)	67 (2.4)	37 (4.9)	
Unknown	74 (2.0)	52 (1.8)	22 (2.9)	
Census tract level poverty				<0.0001
<10%	902 (24.9)	839 (29.4)	63 (8.3)	
10–19.9%	1,407 (38.9)	1,225 (42.9)	182 (23.9)	
≥20%	1,307 (36.2)	790 (27.7)	517 (67.9)	
Census tract level urbanicity				<0.0001
Urban	2,597 (71.8)	1,953 (68.4)	644 (84.5)	
Rural	1,019 (28.2)	901 (31.6)	118 (15.5)	
Time interval from diagnosis to surgery, mean (std)	40.8 (44.8)	32.7 (35.6)	47.1 (43.0)	<0.0001
Time interval from diagnosis to surgery, median (Q1–Q3)	29 (6–50)	27 (3–46)	42 (14–70)	<.0001
Receiving surgery >6 weeks from diagnosis	32.9	28.7	48.4	<0.0001

*AJCC, American Joint Committee on Cancer; std, standard deviation; Q1, the first quartile; Q3, the third quartile*.

Compared to white patients, the OR of receiving surgery more than 6 weeks after diagnosis for black patients was 2.33 (95% confidence interval [CI]: 2.00-2.74) in the crude model, which decreased to 2.19 (95% CI: 1.84–2.60) after controlling demographic and tumor characteristics. With additional adjustment of social determinants, the OR remained significant at 1.91 (95% CI: 1.59–2.30) (Table 2). Being unmarried (OR: 1.27, 95% CI: 1.08–1.48), having Medicare or other public insurance (OR:1.20, 95% CI: 1.01–1.44), having Medicaid insurance (OR:1.67, 95% CI: 1.31–2.14), no insurance (OR:3.03, 95% CI: 1.98–4.65), and living in high poverty level area (OR 1.43, 95% CI: 1.08–1.48) were associated with a significantly higher likelihood of delayed surgery. After stratification by race, living in a high poverty census tract was significantly associated with delayed surgery in both racial groups, but with a higher OR in blacks (OR: 1.91, 95% CI: 1.09–3.35) than in whites (OR: 1.37, 95% CI: 1.10–1.71) (Table 3). Among white patients, the OR of receiving surgery more than 6 weeks after diagnosis was significant for those with Medicare coverage (OR: 2.11, 95% CI: 1.56–2.85) and no insurance coverage (OR: 4.15, 95% CI: 2.46–7.04), compared to those with private insurance coverage. Among black patients, those with Medicaid coverage had a significantly higher risk of delayed surgery (OR: 1.48, 95% CI: 1.01-2.16) (Table 3).

**Table 2:** Odds ratio (95% confidence interval) of receiving surgery 6 weeks or later after diagnosis for non-small cell lung cancer patients.

	Model 1^*^	**Model 2^†^**	**Model 3^¶^**
**Race**			
White	1	1	1
Black	2.33 (2.00, 2.74)	2.19 (1.84, 2.60)	1.91 (1.59, 2.30)
Age, %			
<54		1	1
55–64		1.24 (0.98, 1.58)	1.40 (1.09, 1.80)
65–74		1.14 (0.90,1.43	1.27 (0.98, 1.64)
75+		1.34 (1.03,1.74)	1.48 (1.11, 1.97)
**Sex, %**			
Female		1	1
Male		0.99 (0.86,1.16)	0.93 (0.80, 1.08)
**Tumor histology, %**			
Adenocarcinoma		1	1
Non-adenocarcinoma		1.06 (0.90,1.23)	1.02 (0.87, 1.20)
**Tumor size, %**			
<3 cm		1	1
3–7 cm		1.27 (1.09, 1.49)	1.27 (1.08, 1.49)
>7 cm		0.90 (0.62, 1.29)	0.93 (0.64, 1.34)
**AJCC stage, %**			
Stage I		1	1
Stage II		1.16 (0.89, 1.51)	1.09 (0.84, 1.42)
Stage IIIA		1.46 (1.03, 2.10)	1.43 (0.99, 2.05)
**Lymph node involvement, %**			
Negative		1	1
Positive		0.90 (0.67, 1.20)	0.93 (0.69, 1.25)
**Grade, %**			
Well-differentiated		1	1
Moderately differentiated		1.02 (0.80, 1.31)	0.97 (0.76, 1.25)
Poorly/undifferentiated		1.49 (1.10, 2.10)	1.08 (0.84, 1.40)
**Surgery type, %**			
Resection of lobe		1	1
Resection extended		0.87 (0.69, 1.10)	0.85 (0.67, 1.08)
**Charlson comorbidity index, %**			
0		1	1
1		1.15 (0.98, 1.36)	1.11 (0.94, 1.31)
2+		1.39 (1.14, 1.70)	1.32 (1.08, 1.62)
**Marital status, %**			
Married			1
Single/divorced/widowed/other			1.27 (1.08, 1.48)
Unknown			0.67 (0.41, 1.09)
**Insurance, %**			
Private			1
Medicare/other public			1.20 (1.01, 1.44)
Medicaid			1.67 (1.31, 2.14)
No insurance			3.04 (1.98, 4.65)
Unknown			0.66 (0.34, 1.20)
**Census tract poverty, %**			
<10%			1
10–19.9%			1.17 (0.96, 1.43)
≥20%			1.43 (1.17, 1.76)
**Census tract urbanicity, %**			
Urban			1
Rural			1.02 (0.86, 1.21)

* *Crude model*;

† *Adjusted age at diagnosis, sex, marital status, histology, tumor size, AJCC stage, tumor grade, lymph node involvement status, surgery type, and comorbidity*.

¶ *Additionally adjusted for insurance, marital status, census tract level poverty and urbanicity*.

**Table 3:** Adjusted odds ratios (95% confidence interval) of receiving surgery 6 weeks or later after non-small cell lung cancer diagnosis, stratified by race.

	**White**	**Black**
**Age**
<54	1	1
55–64	1.43 (1.05, 1.94)	1.26 (0.81, 1.96)
65–74	1.22 (0.90, 1.66)	1.39 (0.87, 2.21)
75+	1.64 (1.17, 2.28)	0.92 (0.49, 1.70)
**Sex**
Female	1	1
Male	0.99 (0.83, 1.18)	0.76 (0.55, 1.05)
**Tumor histology**
Adenocarcinoma	1	1
Non-adenocarcinoma	1.12 (0.94, 1.34)	0.74 (0.53, 1.03)
**AJCC Stage**
Stage I	1	1
Stage II	1.16 (0.85, 1.59)	0.90 (0.53, 1.03)
Stage IIIA	1.43 (0.93, 2.19)	1.36 (0.69, 2.71)
**Tumor size**
<3cm	1	1
3–7cm	1.26 (1.05, 1.52)	1.30 (0.93, 1.81)
>7cm	0.96 (0.63, 1.47)	0.80 (0.39, 1.66)
**Lymph node involvement**
Negative	1	1
Positive	0.96 (0.68, 1.35)	0.82 (0.47, 1.44)
**Grade**
Well-differentiated	1	1
Moderately differentiated	0.99 (0.74, 1.33)	0.84 (0.50, 1.40)
Poorly/undifferentiated	1.10 (0.80, 1.47)	0.98 (0.57, 1.66)
**Surgery type**
Resection of lobe	1	1
Resection extended	0.90 (0.68, 1.18)	0.72 (0.44, 1.17)
**Comorbidity index**
0	1	1
1	1.11 (0.91, 1.34)	1.13 (0.80, 1.61)
2+	1.33 (1.05, 1.70)	1.29 (0.86, 1.94)
**Marital status**
Married	1	1
Single/divorced/widowed/other	1.26 (1.05, 1.52)	1.21 (0.87, 1.68)
**Insurance**
Private	1	1
Medicare/other public	1.123 (0.92, 1.38)	1.47 (1.01, 2.15)
Medicaid	2.13 (1.57,2.88)	1.16 (0.75, 1.79)
No insurance	4.10 (2.42, 6.95)	1.55 (0.75, 3.21)
Unknown	0.67 (0.31,1.44)	0.74 (0.28, 1.94)
**Census tract poverty**
<10%	1	1
10–19.9%	1.14 (0.92, 1.40)	1.49 (0.80, 2.75)
≥20%	1.37 (1.10, 1.72)	1.90 (1.08, 3.34)
**Census tract urbanicity**
Urban	1	1
Rural	0.97 (0.81, 1.17)	1.33 (0.87, 2.02)

From the mediation analysis, the total effect of race on timely surgery was 0.905 (95% CI; 0.735–1.091), the direct effect was 0.652 (95% CI: 0.470–0.854), and the indirect effect through clinical factors and social determinants were 0.009 (95% CI: −0.013–0.028), and 0.235 (95% CI: 0.151–0.321). The percentage of racial disparities transmitted through social determinants was 25.97%.

## Discussion

With population-based data collected by the state cancer registry, we found that black stage I-IIIA NSCLC patients had almost two-fold odds of receiving delayed surgery, compared to their white counterparts. Multiple social determinants, including insurance, marital status, and poverty level in the census tract, were significant predictors of delayed surgery, but these factors did not fully explain the racial disparity in delayed NSCLC surgery in a U.S. southern state.

Our findings of a higher risk of delayed surgery among black patients are consistent with a few studies using nationwide databases. However, the magnitude of the racial disparity was more profound in Louisiana than in U.S. on average. One recent study using 2008–2013 National Cancer Data Base (NCDB) reported a median time from diagnosis to surgical resection of 26 days for whites and 31 days for blacks (*p* < 0.0001) (9). White patients in Louisiana had a similar median wait time for surgery compared to white patients in the U.S. (27 days vs. 26 days), but black patients in Louisiana experienced a 30% longer wait time compared to black patients in the U.S. (42 days vs. 31 days). The larger difference in diagnosis-to-surgery interval between black and white patients in Louisiana is also reflected in the higher OR found in our study. One study using SEER-Medicare data reported that black NSCLC patients had 1.18 times the odds of receiving delayed treatment compared to white patients (23). Another study using NCDB data reported an adjusted OR of 1.48 of delayed surgery in blacks than in whites. In Louisiana, black patients had 1.92 times the odds of having delay in NSCLC surgery compared to white patients, after adjusting for clinical factors and social determinants.

We examined the impact from several social determinants on timely surgery and whether the examined social determinants could explain the observed racial disparity. Unsurprisingly, private insurance was a significant predictor of the receipt of timely surgery or guideline-concordant care in lung cancer patients (17). Medicaid insured patients tended to experience the largest delay in receiving treatment (17). In a national study, compared to NSCLC patients with private insurance, the diagnosis-to-surgery interval was 2.3 days longer for Medicare patients, 10.8 days longer for Medicaid patients, and 7.8 days for longer for patients who were uninsured or whose insurance status was unknown (9). Another study found that patients covered by both Medicare and Medicaid were less likely to receive timely surgical treatment than patients with only Medicare (23). Insurance was also a significant predictor of delayed surgery in our study, however, we additionally found different effects of insurance coverage on timely treatment in two racial groups. Among white patients, Medicaid covered patients had two times the odds and patients without insurance had four times the odds of having delayed surgery compared to their privately insured counterparts. Compared to black patients with private insurance, the odds of receiving surgery more than 6 weeks after diagnosis was 1.5 times for the Medicare group, but no significant differences were observed for those with Medicaid or those without insurance. The small sample size of black patients may be a reason for a lack of finding significant ORs. However, the point estimates of the ORs in these two groups were also lower in black patients than in white patients. Another possible reason is the availability of charity hospitals in Louisiana, which provide medical services to uninsured residents. As the majority of patients receiving care through charity hospitals are black, the black uninsured patients may have had more healthcare access benefits through the charity hospitals than white uninsured patients. This could be a possible explanation of the insignificant odds ratios of receiving delayed surgery for black Medicaid covered or uninsured patients compared to black privately insured patients (in contrast with the highly significant odds ratios observed in white patients). The adoption of public policies, such as the Affordable Care Act (ACA) can improve access to high quality healthcare for marginalized populations receiving disparate care. Previous studies have shown that cancer survivors in Medicaid expansion states were more likely to be insured, to have access to care, and to be diagnosed at an early stage of disease than those in non-expansion states (17, 24). Louisiana was one of the states with earliest initiation of ACA expansion in the country (23).

Our findings indicate that those who are single, widowed or divorced are more likely to receive delayed surgical treatment for NSCLC. Being married or living with a partner is an indication of social support. Social support has been found to be a protective factor for the prevention and maintenance of many diseases (25). Previous studies reported that being married is a predictor of receiving standard care and better outcomes (26–28), while experiencing social isolation or loneliness has a negative influence on lung cancer patients (29). In our study, being married showed a similar protective effect for receiving timely surgery in both white and black patients, while the insignificance of the OR among black patients may be due to the smaller sample size of black patients in our study.

Our study revealed that living in census tracts with a higher proportion of the population living above the national poverty level was associated with higher risk of receiving delayed surgery, and the relationship showed a dose-response effect. This finding is consistent with previous research that evaluated both individual level household income and census-tract level income (9, 30). Even among Medicare covered NSCLC patients who have equal health care access, income level is positively associated with timely treatment (23). After stratification by race, we found that the impact from income was even stronger among black patients, while the strength of the association among white patients was similar to findings from national data (23). Although urban residence was associated with lower lung cancer incidence and better outcomes (31, 32), similar to another study (23), living in urban census tracts was not a significant predictor of timely surgery in our study.

Unsurprisingly, social determinants of health are important contributing factors of racial disparities in cancer prognosis and outcomes (33–35). It has also been found that social determinants account for racial disparities in receiving guideline-recommended curative treatment for NSCLC, breast, and prostate cancer, specifically with insurance status, geographical access, and SES factors having been identified as contributors (36–39). Although black NSCLC patients are less likely to receive guideline concordant surgical treatment than whites, the literature on the role of social determinants in the receipt of timely cancer was limited (27, 40, 41). Black women are less likely on average to receive hormone therapy, chemotherapy, radiotherapy and surgical treatment for breast cancer in a timely manner as compared to white women (42). Additionally, both high and low-risk black prostate cancer patients experienced longer wait times from diagnosis to definitive treatment compared to white patients (43). One study reported that the differences in social determinants explained about 26% of racial disparities, while in our study, the majority of the racial disparity was not explained by the measured mediators. The remaining unexplained racial disparity indicates that there are other social-cultural differences between the two racial groups that influence the timeliness of surgical receipt for NSCLC. One limitation of our study is the lack of information on patients' perceptions and attitudes toward cancer treatment. Black patients may be reluctant to seek care due to stigma, distrust in physicians, and negative perceptions about surgery. Previous research indicates that black, at-risk NSCLC patients commonly seek a second opinion or are skeptical of information provided to them by a physician with whom they had no previous relationship with (44). Black patients also tend to have negative perceptions about surgical treatment, and believe it to be riskier than radiation or chemotherapy treatment (45). Future research could examine whether these risk factors can explain racial disparity in timely NSCLC surgery and design tailored intervention to improve the timely treatment in both racial groups. Another limitation of this study is that we did not have data on several individual-level social determinants, such as household income, education, and employment. Lower educational level is associated with decreased odds of having surgery and poorer survival rates (32). Population-based administrative data or cancer registry data usually do not have information on such individual-level variables. Surveys or additional medical record data extraction are needed to address this gap. Despite these limitations, our study is the first to investigate the impact of social determinants of health on receipt of timely NSCLC surgical treatment by race. Our findings can provide important evidence for future intervention.

In summary, while timely surgery is an important predictor of the prognosis of curable NSCLC, we found a significant racial disparity among Louisiana patients. Black patients had almost twice the odds of receiving delayed surgery than white patients, after adjusting for demographic, clinical, and social factors. Having social support, private insurance, and living in census tracts with higher income level was associated with improved access to timely surgery, but these factors explained only about 26% of the observed racial disparity. Majority of racial disparity remained unexplained. As the state of Louisiana has high proportion of black population and high lung cancer incidence, the findings from this study provide evidence for tailored interventions to improve timely treatment. Black patients living in census tracts with a higher poverty level should be particularly targeted, as they experienced a higher risk of delayed surgery. Future studies are needed to examine the effects of other individual level social determinants to decipher the racial disparity in NSCLC timely treatment.

## Data Availability Statement

The data analyzed in this study is subject to the following licenses/restrictions: The de-identified dataset can be requested through the Louisiana Tumor Registry. Requests to access these datasets should be directed to mhsieh@lsuhsc.edu.

## Author Contributions

M-CH and X-CW provided data. M-CH and LZ ran the analyses. PN and LZ wrote the first draft of the paper, which was reviewed by all authors. All authors conceptualized, initiated the study and provided advice.

## Conflict of Interest

The authors declare that the research was conducted in the absence of any commercial or financial relationships that could be construed as a potential conflict of interest.
